# Associations between caregiver mental health and young children’s behaviour in a rural Kenyan sample

**DOI:** 10.1080/16549716.2020.1861909

**Published:** 2021-01-04

**Authors:** Christina A. Laurenzi, Xanthe Hunt, Sarah Skeen, Phillip Sundin, Robert E. Weiss, Victor Kosi, Mary Jane Rotheram-Borus, Mark Tomlinson

**Affiliations:** a Institute for Life Course Health Research, Department of Global Health, Faculty of Medicine and Health Sciences, Stellenbosch University, Stellenbosch, South Africa; b Department of Biostatistics, Fielding School of Public Health, University of California, Los Angeles, CA, USA; c Plan International, Kisumu, Kenya; d School of Nursing and Midwifery, Queens University, Belfast, UK

**Keywords:** Child behaviour, caregiver depression, caregiver anxiety, parenting stress, Kenya, Sub-Saharan Africa

## Abstract

**Background**: Research shows that caregiver mental health problems have direct, significant effects on child behaviour. While these risks are amplified in low-resource settings, limited evidence exists from these places, especially sub-Saharan Africa.

**Objective**: We measured associations between caregiver mental health and child behaviour in a rural Kenyan sample, hypothesizing that higher rates of caregiver mental health would be associated with increased child behavioural problems. We also sought to provide an overview of caregiver mental health symptoms in our sample.

**Method**: Cross-sectional data were collected from caregivers of children ages 4–5 years old enrolled in a community-based early child development programme in western Kenya. 465 caregivers were recruited and assessed at baseline, and answered questions about child behaviour, mental health symptoms (depression, anxiety, stress), and help-seeking. A multivariate linear regression model was used to assess significance of each mental health factor.

**Results**: Caregiver anxiety (*p* = 0.01) and parenting stress (*p* < 0.001) were significantly associated with child behavioural problems. 245 caregivers (52.9%) had high levels of symptoms of depression, anxiety, or both; furthermore, 101 caregivers (21.7%) scored above the cut-off for both of these scales. A high proportion of our sample (60.6%) reported seeking some formal or informal psychosocial support services; however, less than one-third of these caregivers were symptomatic (30.9%).

**Conclusion**: Anxiety and stress were associated with poorer child behavioural outcomes. Our sample reflected a higher prevalence of caregiving adults with mental health symptomology than previous estimates from Kenya, with few high-symptom caregivers seeking support. We discuss further implications for programming and health services delivery.

## Background

There has been a renewed emphasis on mental health within the global development agenda, as emerging evidence points to a high burden of disease in low-resource settings [1]. However, there remains an uneven distribution of research, resources, and practitioners to fully address mental health problems in low- and middle-income countries (LMICs) [2]. These limitations have led to gaps in understanding the effects of mental health across the life course, including during early childhood. A growing body of literature is now exploring how mental health affects child development in LMICs, especially among young children [3–6].

Caregiver mental wellbeing is important for ensuring healthy child behavioural outcomes and development. Caregivers may refer to the child’s biological parents but also includes any primary adult responsible for the child. Responsive caregiving, identified in the Nurturing Care Framework, is prioritised as one of five core domains contributing to optimal early child development [7]. However, many caregivers struggling with mental health challenges are unable to be sensitive and responsive; these problems can greatly interfere with caregiver responsibilities [8–10]. Previous research shows that caregiver mental ill health has direct, significant effects on child behaviour [11]. Researchers have established links between perinatal maternal depression and later instances of parenting stress [12,13]. Evidence also shows additive effects of parental depression and anxiety on child behavioural problems [14], as well as longer-term associations between maternal mental health and child behaviour [9,15]. Children whose caregivers have mental health conditions – and may be less attentive and sensitive – may be at risk for developing mental health conditions themselves, or may struggle with typical social and behavioural development [9,16,17]. Mutually, mothers distress may increase child behaviour problems or children’s behaviour problems may influence child distress [18].

Across LMICs in particular, chronic poverty and economic stress can exacerbate adult depression, anxiety, stress, and substance use, further limiting an individual’s or household’s ability to function, and in turn, affecting child wellbeing [19]. Gender inequality and interpersonal violence can contribute to maternal mental health problems and parenting challenges [20], and these phenomena are prevalent across LMIC settings [21]. These risk factors, sometimes referred to as ‘accumulated adversities’, in turn affect child development and behaviour [22]. Children living in adverse conditions are more prone to developing mental health disorders of their own [19,23,24]. Furthermore, in sub-Saharan African countries bearing a disproportionate burden of the global HIV/AIDS epidemic, evidence has found that the burden of disease on families and households can pose a special risk for child and adolescent mental health [25–28].

Despite the multiple risks to child wellbeing in low-resource contexts, much of the research in LMICs on adult mental health and its link to child behaviour has thus far focused on the role of caregiver depression [29,30] as well as earlier effects of perinatal mental health problems [31–34].

In this paper, we set out to examine associations between caregiver mental health and child behaviour in a rural district in Kenya. As a secondary aim, we also sought to document rates of caregiver depression, anxiety, and parenting stress. Few studies assess parenting stress and child wellbeing in LMICs, including East African countries [3,35,36], and there is limited research documenting parental stress and anxiety alongside depression in LMICs, specifically exploring if poor parental mental health across the spectrum can predict child behavioural problems. As more efforts are being made worldwide to reduce the burden of non-communicable diseases including mental health disorders, this is a missing link between mental health and early childhood development, one that merits further exploration.

## Methods

### Design

We collected baseline cross-sectional data from caregivers of children enrolled in Plan International’s Community-Led Action for Children (CLAC) programme. Plan International is an international non-governmental organisation that develops and implements programmes on child rights, health, and education, operating in 75 countries worldwide, and 25 in sub-Saharan Africa. The study has ethical approval from the ethics boards at Stellenbosch University (N15/10/099) and the Ethics & Scientific Review Committee at AMREF in Kenya (P220/2016).

### Setting

The study took place in Nyanza region of Kenya, in predominantly rural Kisumu, Homa Bay, and Siaya Districts. Little regionalised data exist on adult mental health. Caregivers of children ages 4–5 were recruited through their child’s attendance at early childhood care and development (ECCD) centres. These ECCD centres are typically part of government primary schools. At the time of the study’s initiation in 2015, Plan was providing support for 99 ECCD centres across Nyanza, expanding learning capacity and facilitating centre-affiliated parenting groups open to all caregivers.

### Sample

Plan staff in Kenya generated a list of all CLAC-supported centres, assigning each centre a rating of low (1), average (2), or high (3) quality across four domains: quality of parent management committee, teacher competence, facilities (water, sanitation, structure), and equipment (for teaching, e.g. toys per child). Sum scores (range = 4–12) dictated whether an ECCD centre fell into a low (4–6), average (7–9), or high (10–12) quality category. We selected 20 ECCD centres, roughly equally distributed across all three quality levels and regions, as recruitment sites for the study. Quality ratings were used to ensure a diversity of school environments to consider alongside other factors linked to child development and child mental health for other research questions explored in this study. We later dropped one of these centres after an incident of community violence.

Caregivers were eligible for recruitment if at least one child was attending one of these selected ECCD centres, and if the child was four or five years of age at the time of the baseline recruitment. The study team recruited, on average, 30 caregivers per centre. In those centres with less than 30 eligible caregivers, all were included; where there were more than 30, the team selected every third name from a full roster of eligible-aged children until the target number was reached. Caregivers had to consent to be interviewed before being enrolled in the study.

### Data collection procedure

We employed and trained a team of experienced data collectors from the study region to interview caregivers about themselves and their children. The team, with guidance from Plan Kenya’s staff and programme facilitators, held informational meetings for caregivers and teachers ahead of data collection at each school.

Prior to each interview, data collectors administered informed consent to each caregiver, and allowed caregivers to ask questions or raise concerns. Once enrolled into the study, each caregiver was assigned a randomly generated participant identifier to anonymise their data. All consent forms were securely stored with the on-site supervisor and transferred to Stellenbosch University at a later date.

All standardised scales used in the questionnaires were translated independently by two members of the Kenyan research team into Luo and Swahili, and assessed for cultural relevance as well as local understanding. Back translations provided additional rigour. No changes to the scales or items were made; however, the team discussed ways to further explain items that might be confusing to participants, to ensure a level of rigour across data collection.

Questionnaires were loaded onto a tablet-based research application. The data collection team, fluent in both languages, conducted all interviews in the caregiver’s preferred language. Interviews were arranged ahead of time, and conducted at a neutral location close to the ECCD centre, typically an empty church hall. Data collectors entered all questionnaire responses into the application; when completed, these interviews were submitted and securely stored in an online database accessible only by project managers. Data collection via mobile device is a proven method to improve data security in remote field sites, ensure efficient data collection, and protect against accidental data loss [37].

## Measures

Caregivers were interviewed using a standardised questionnaire with two parts; one about the caregiver and household, and one about the child.

### Demographics

We asked demographic questions about the caregiver, household, and child. Questions about caregivers included gender; age; highest level of education completed; marital status (including polygamous union); and HIV status. Questions about the household included water source; electricity source; household assets index; household income sources; and number of household members. Questions pertaining to the child included child gender; age; relationship to caregiver; number of siblings; whether the child was ever left home alone; who was responsible for disciplining the child (male or female caregivers); and child HIV status. The region where the child attended an ECCD programme was also included.

### Caregiver mental health measures

We used the Patient Health Questionnaire-9 (PHQ-9) to gauge depression among our sample of caregivers [38]. The 9-item scale identifies specific physical and emotional symptoms; interviewers ask caregivers to rate the frequency with which they have experienced a given symptom over the past two weeks. Caregivers indicate if they experienced the feeling never, a few days, more than half the days, or nearly every day. It has been validated and used within sub-Saharan countries, including Ethiopia [39] and Kenya [40]. A cutoff score of 10 was chosen based on work by Gelaye et al. which identified a sensitivity of 86% and specificity of 67% in establishing the likelihood of major depressive disorder in an East African population [39].

We used the Generalised Anxiety Disorders-7 (GAD-7) to gauge levels of anxiety among caregivers [41]. The 7-item scale uses the same response scale as the PHQ-9. It has been used across sub-Saharan Africa in countries such as Ghana, Cote d’Ivoire [42], and Lesotho [43]. As with the PHQ-9, a cutoff score on 10 has been validated by other research with a sensitivity of 76% and a specificity of 64% [44].

We also included the Parenting Distress subset from the Parenting Stress Index-Short Form (PSI-SF). This subscale comprises 12 items, and is used to gauge stress related to parenting responsibilities, difficulties, and life changes [45]. Interviewers prompt caregivers to respond to a statement with the following options: strongly disagree, disagree, neither agree nor disagree, agree, strongly agree. Strong agreement indicates higher levels of distress. This scale has been used in South Africa [46], Ghana and Cote D’Ivoire [47], and Kenya [35].

We included one question to further gauge mental health, inquiring about the participant’s experience seeking either formal or informal services for support: ‘in the last year, have you felt so emotionally or spiritually troubled that you felt you needed to consult a healer (spiritual healer, faith healer, or traditional healer), counsellor or health worker (clinic nurse or doctor)?’

### Child behavioural outcomes

We collected parent-reported data on child behaviour with the Strengths and Difficulties Questionnaire (SDQ), designed for use with 4–17 year-old children [48]. Caregivers were prompted with a specific behaviour and responded if it was not true, somewhat true, or certainly true. The set of 25 questions is grouped into internalising and externalising scales, and 20 of these questions are combined to produce a total difficulties score. This measure has been used in high-income settings to analyse parental mental health and child behaviour [15], and has been used in some sub-Saharan African countries [49,50]. In Kenya, it has also been used with adolescents [51].

## Statistical methods

The primary outcome variable was child behaviour measured by the SDQ (using the total difficulty score). Children, not caregivers, were the unit of analysis; if a caregiver had multiple children, one child was selected at random. Each child had an equal chance of being selected for the analysis, and results were run with different combinations of selected children. Results were similar (see Table S1, supplementary material), and thus we report results for the whole sample. Univariate linear regressions were used to identify demographics highly correlated with caregiver mental health. Any demographic variable that was significant for predicting at least two out of the four caregiver mental health was used in a multivariate linear regression model with all four caregiver mental health variables to test for predictors of child behavioural outcomes (see Table S2, supplementary material). We considered a random caregiver effect, but it was not significant nor did it affect results: those analyses are omitted. All analyses were done in R [52].

## Results

We analysed data from 465 caregivers and 497 children; 32 caregivers were responsible for two children. There were 239 (48%) female children, whereas 94% of caregivers were female. Most caregivers (72%) were the child’s biological mother. Full demographic characteristics are shown in Table 1.

**Table 1.** Demographic information (N = 497)

	N (child)	%
Caregiver education		
No School	18	3.62%
Grades 1–6	105	21.13%
Grades 7–8	267	53.72%
Grades 9–10	33	6.64%
Grades 11–12	51	10.26%
Some secondary education	23	4.63%
Caregiver gender		
Male	59	11.87%
Female	438	88.13%
Caregiver marital status		
Unmarried	38	7.65%
Married		
Monogamous union	337	67.81%
Polygamous union	122	24.55%
Caregiver relation to child		
Biological mother	357	71.83%
Biological father	51	10.26%
Grandmother	56	11.27%
Other relative	33	6.64%
Household members		
0–3	55	11.07%
5-Apr	210	42.25%
7-Jun	154	30.99%
8+	78	15.69%
Household monthly income		
1 (0–2000 Ksh)	291	58.55%
2 (2001–5000 Ksh)	108	21.73%
3 (greater than 5000)	51	10.26%
Do not know/decline to answer	47	9.46%
Child Gender		
Male	258	51.91%
Female	239	48.09%
Child age (months)		
48–59	268	53.92%
60–71	229	46.08%
Do you ever leave child home alone?		
Yes	168	33.80%
No	329	66.20%
Water source		
Water on premises	21	4.23%
Water from community tap/public tank/well	246	49.50%
Water from a river	230	46.28%
Region		
Bondo	172	34.61%
Homa Bay	190	38.23%
Kisumu	135	27.16%
Caregiver HIV status		
Positive	111	22.33%
Negative	365	73.44%
NA	21	4.23%
Child HIV status		
Positive	8	1.61%
Negative	396	79.68%
NA	93	18.71%
Electricity		
Has electricity	63	12.68%
Does not have electricity	434	87.32%
Household items		
Stove	133	26.76%
Cell phone	474	95.37%
Bicycle	169	34.00%
Radio	338	68.01%
Television	55	11.07%
Internet via phone	114	22.94%
Household income source		
Earned income	470	94.57%
Community savings funds	1^85^	37.22%
Other (grants, pensions, child support)	38	7.65%
SDQ Scores	Mean = 12.74	Standard dev = 5.58

There were 196 caregivers (42.15%) who scored 10 or higher on the PHQ-9, and 50 caregivers (32.26%) scored 10 or higher on the GAD-7. Together, 101 caregivers (21.72%) scored 10 or higher on both scales (Table 2).

**Table 2.** Prevalence of caregiver mental health symptoms

	Anxiety GAD-7 < 10 (% of total)	Anxiety GAD-7 ≥ 10 (% of total)	Sum (% of total)
Depression PHQ-9 < 10	220 (47.31%)	49 (10.54%)	269 (57.85%)
Depression PHQ-9 ≥ 10	95 (20.43%)	101 (21.72%)	196 (42.15%)
Sum (% of total)	315 (67.74%)	150 (32.36%)	465

Altogether, 245 (52.5%) caregivers had high probability of experiencing above-cutoff levels of depression, anxiety, or both. Furthermore, 282 caregivers (60.64%) reported seeking informal or formal support for emotional trouble in the past year. Of this support-seeking group, 87 (30.9%) caregivers were symptomatic (PHQ-9 or GAD-7 score of 10 or higher). Conversely, about one-third (35.5%) of symptomatic caregivers reported seeking some form of support in the past year.

In univariate analyses, we use raw SDQ scores instead of cutoff values, which enable the use of linear regression modelling. Both depression and anxiety had a positive association with child behaviour (SDQ), as increased caregiver depression and anxiety scores are associated with higher SDQ scores and therefore worse child outcomes. Parenting stress and informal or formal support-seeking were significantly negatively correlated with SDQ scores (Table 3). Based on the coding of these variables, this indicates that higher parenting stress and not seeking support are associated with higher SDQ scores.

**Table 3.** Depression variables regressed on child aggregate outcomes (univariate regression)

Outcome	Predictor	Estimate	Std Error	t-Value	p-Value
Child behaviour (SDQ, total difficulties score)	Anxiety (GAD-7)	0.31	0.05	6.01	<0.001
	Depression (PHQ-9)	0.26	0.05	5.23	<0.001
	Informal or formal support sought	−1.28	0.51	−2.52	0.01
	Parenting stress (PSI)	−0.19	0.03	−7.08	<0.001

In our univariate analyses, which tested the demographic predictors shown in Table 1, five demographic predictors had a significant positive association for two of the four caregiver mental health outcomes: having electricity; household possession of a cell phone, radio, or television; and ever leaving a child home alone. Gender was not found to be significant for either child or caregiver. Number of household members and child number of siblings negatively predicted caregiver mental health (see Table S2, supplementary material).

Table 4 shows results from the multivariable regression model predicting SDQ score. Two mental health variables significantly predicted child outcomes. Ever leaving a child home alone was also significant when controlling for other demographic variables, and informal or formal support-seeking was not significant in the multivariable model.

**Table 4.** Multivariate linear regression with child behaviour (SDQ, total difficulties score) as outcome

Predictor	Estimate	Std. Error	t-Value	p-Value
Electricity	0.61	0.76	0.81	0.42
Cell phone ownership	−1.44	1.13	−1.28	0.20
Radio ownership	0.66	0.51	1.29	0.20
Television ownership	0.39	0.81	0.48	0.63
Number of household members	0.01	0.14	0.05	0.96
Child ever left home alone	−1.62	0.50	−3.26	0.001**
Number of siblings	−0.34	0.14	−2.32	0.02**
Anxiety (GAD-7)	0.17	0.06	2.68	0.01**
Depression (PHQ-9)	0.09	0.06	1.35	0.18
Parenting stress (PSI)	−0.14	0.03	−5.13	<0.001**
Informal or formal support sought	−0.71	0.49	−1.44	0.15

**p < 0.05.

## Discussion

This study investigates the relationship between caregiver mental health and child behaviour, measuring outcomes of preschool-aged children in a rural Kenyan sample. To our knowledge, this analysis is the first to do so within this population. We found high levels of symptoms of depression and anxiety experienced by caregivers, which predicted child behaviour problems.

We found a strong predictive effect of caregiver mental health status on child behavioural outcomes, specifically that anxiety and stress were associated with poorer child behavioural outcomes. One comparable study from a rural Ethiopian sample found similar associations to ours, with parental mental disorders predicting child developmental outcomes of children under the age of two [3]. While other studies from LMICs and Kenya have identified caregiver depression as a significant predictor of child outcomes [53–55], we did not identify depressive symptoms as significant in our multivariate analysis. Instead, we found that caregiver anxiety had a larger effect size in our multivariate model than caregiver depression for predicting poor child behavioural outcomes. Some prior research has attempted to look at differential effects of depression and anxiety in caregiver–child relationships [56]. Other studies have isolated parental anxiety in analysing child behavioural outcomes; Murray et. al. [57] found that maternal anxiety affected parenting behaviours, particularly in high-stress situations. Martini et. al. [58] examined anxiety during pregnancy among a cohort of German mothers, and found that anxiety disorders, and not externalising disorders, were likely to be passed on to children. However, in our review of the literature, similar findings have not been replicated in other LMICs.

The impact of parenting stress on children has mixed evidence from other studies. Allen and colleagues [25] found that higher levels of parenting stress predicted poorer child mental health outcomes, and more specifically, lower adaptive functioning in communication and daily living skills. Oburu (2005), however, who also looked at child outcomes using the SDQ scale in a Nyanza-based sample of orphaned children, found no correlation between elevated stress of grandparent caregivers and child outcomes. Parenting stress may be more closely related to child behavioural outcomes than some of these studies have found.

With regard to our secondary focus, we also found high levels of caregiver mental health symptoms. Just over half of our sample reported symptoms consistent with depression, anxiety, or both, indicating a high prevalence of adult mental health problems in the communities surveyed. This prevalence is higher than other supporting evidence suggests; the most recent Kenyan WHO Global Health Estimates are low for depressive (4.4%) and anxiety (3.1%) disorders [59]. Another survey from Nyanza Province found an adult prevalence of 10.8% for common mental disorders, taken from a random household sample [36]. It may be possible that by sampling caregivers with caregiving responsibilities for young children, mental health trends may be overestimated in our sample; however, our caregivers also represented a diversity of educational, gender, and economic characteristics, which may similarly affect their mental health. Other prevalence studies have found similarly high levels of depression, anxiety, and stress in high-HIV burden populations, including South Africa [60,61], Zimbabwe [62] and Tanzania [63]. Although our study does not provide diagnostic criteria for common mental disorders, the PHQ-9, for instance has been found to provide an appropriate screening for major depression disorder [64], and as such we believe that the tools used can help approximate how levels within our sample compare to existing nation-wide mental health trends.

Although a high proportion of our sample reported informal or formal support-seeking in the past year, there was little overlap between this group and the group found to be experiencing mental health problems. That only one-third of the caregiver sample showing symptoms of depression and/or anxiety reported seeking support likely indicates stigma or challenges surrounding help-seeking – something that has been established among adults with mental health problems in other African settings [65–67].

Finally, we found two home environment-related variables that are worth exploring further. Number of siblings and child behavioural outcomes had an inverse relationship, indicating that children with a greater number of siblings were reported to have fewer behavioural problems. This trend has been identified in other research including from Brazil [68] and Norway [69] but may require further investigation in this specific setting to understand mechanisms by which this association may work. We also found that caregivers who ever left their children home alone also reported more child behavioural problems, which may have to do with less time under adult supervision or structured care. Notably, children being left home alone did not correlate with caregiver depression or household earned income. Ruiz-Casares and Heymann explored the prevalence of leaving children home alone across Botswana, Mexico, and Vietnam, and found high rates of this practice in Botswana (around one-half of their sample) [70]. There is little research from LMICs about the effects of this practice on child behaviour, although it may be a function of resource constraints or accepted practice; however, there is a significant body of research on the risk factors and harmful effects of child neglect in high-income settings [71,72]. Further research on links between caregiver mental health status, household structure and practices, and child care in LMICs is needed.

### Implications

These findings add to a limited knowledge base about types of mental health disorders prevalent in the Kenyan population, as well as the sociodemographic and caregiver mental health factors that most significantly affect child wellbeing. Our results emphasise the importance of accounting for mental health within LMIC health initiatives at district and local levels. Recent research on mental health in Kenya identifies a high prevalence of mental health problems across the population [40,73,74]. At the same time, capacity to manage these disorders is limited, and institutions suffer from tremendous human resource shortages as well as financial scarcity, overburdening primary care systems [75,76].

While Kenya’s National Mental Health Plan 2015–2030 represents an important collaborative effort with the World Health Organization to address mental health problems and invest in resources, technologies, and infrastructure to increase access to care, disparities still exist [77]. Strong community-based solutions can extend these gains to the most vulnerable citizens, and local initiatives can be strategically deployed to support individuals and community in more holistic ways. Vally and Abrahams [78] explore approaches for peer-delivered mental health services, which is a potentially attractive option in LMIC settings with limited mental health infrastructure. Community-based organizations, such as Plan, can further buffer some of these challenges in developing and implementing programming that supports parents in their roles and connects them with community resources.

However, our findings must be seen within the context of a growing body of literature which suggests that intervening with parents to improve their mental health alone may not necessarily affect child outcomes [6,79]. Efforts must be made to identify ways in which mental health can be addressed in combination with other programmatic elements in a manner which *does* lead to improvements in child wellbeing. Parenting education and support groups are one specific mode of service delivery and capacity development that can enable parents to confront issues of adult and child mental health alongside other related child development issues. Innovative work is currently being done in respect of so-called Sustainable Development Goal (SDG) accelerators; interventions which maximise progress towards SDG-aligned targets, including those related to child development [80]. It may be necessary, then, for programming aimed at supporting child development in the context of poor caregiver mental health need to address one or more of these progress accelerators (government cash transfers to households, safe schools, free schools, parenting support, free school meals, and support groups), alongside mental health programming, in order to maximise the opportunity for effects of child development.

### Limitations

This study has some limitations. Our sample only includes children attending government-funded ECCD centres and their caregivers; thus, it may exclude the most vulnerable families in given communities, as well as children in private ECCD centres. UNICEF estimates for pre-primary school participation in Kenya are around 52% for the period between 2008 and 2012 [81]; however, rates of public pre-primary school uptake tend to be higher than for private schools in rural settings [82]. Furthermore, few child development measures have been developed for LMIC contexts [83]. While we chose measures that we deemed appropriate and that have been used in these contexts [84], many of our measures have not been validated locally. All measures were self-report, and child outcomes were parent-report, allowing for the possibility of reporting bias which may have also been affected by parent anxiety or other barriers. Regarding statistical approach used, our one-step forward selection method for including covariates has a lower type I error than the nominal alpha = 0.05 and thus is conservative: it is less likely to incorrectly claim significance for included variables than a single test of significance. One final note is that our focus on caregiver mental health is skewed towards mothers and other female primary caregivers, as male primary caregivers were less commonly interviewed in our sample.

## Conclusion

Caregiver mental health has significant effects on the wellbeing and behavioural development of their children. Our findings from a rural Kenyan sample show that these effects are significant in LMICs and rural communities, and indicate that there is more research to be done in LMICs on the link between caregiver and child wellbeing.

## Supplementary Material

Supplemental Material

## Paper context

While the link between caregiver mental health and child wellbeing has been documented in high-resource settings, less is known about this association in lower-resource settings. This paper adds important evidence from a cross-sectional sample of caregivers and children in Kenya, showing that high levels of anxiety and depressive symptoms are associated with poorer child behavioural outcomes. These findings emphasize the need for community-driven programming that fully addresses mental health while supporting caregiving education and skills.
