# Supplementary material for: Associations between caregiver mental health and young children’s behaviour in a rural Kenyan sample
Source: Glob Health Action. 2021 Jan 4;14(1):1861909. doi: 10.1080/16549716.2020.1861909 (PMC7801103; doi:10.1080/16549716.2020.1861909)
Supplement: Supplemental Material [file ZGHA_A_1861909_SM2488.docx]

**Title: Associations between caregiver mental health and young children’s behaviour in a rural Kenyan sample**

**Supplementary material**

**Table S1: Multiple regression on randomly selected child (n=465)**

| **Predictor** | **Estimate** | **Std. Error** | **t-Value** | **p-Value** |
| --- | --- | --- | --- | --- |
| Electricity | 0.51 | 0.80 | 0.64 | 0.52 |
| Cell phone ownership | -1.59 | 1.20 | -1.32 | 0.19 |
| Radio ownership | 0.60 | 0.53 | 1.15 | 0.25 |
| Television ownership | 0.31 | 0.84 | 0.37 | 0.71 |
| Number of household members | 0.02 | 0.14 | 0.13 | 0.90 |
| Child ever left home alone | -1.38 | 0.51 | -2.70 | 0.01* |
| Number of siblings | -0.33 | 0.15 | -2.18 | 0.03* |
| Anxiety (GAD-7) | 0.17 | 0.07 | 2.54 | 0.01* |
| Depression (PHQ-9) | 0.07 | 0.07 | 1.00 | 0.32 |
| Parenting stress (PSI) | -0.15 | 0.03 | -5.07 | <0.001* |
| Informal or formal support sought | -0.99 | 0.51 | -1.94 | 0.05* |

**Note:** N=32 caregivers had two children involved in the study, one of whom was chosen at random for inclusion in the anaylses. This table has been included to show the similarity between the findings reported in the main manuscript and those which would have been reported had a separate child under each caregiver been selected at random. Please see the Statistical Methods section of the paper for a detailed description of this process.

**Table S2: Univariate Regression P-values on Depression Outcomes**

| **Demographic** | **Anxiety (GAD-7)** | **Depression (PHQ-9)** | **Informal or formal support sought** | **Parenting stress (PSI)** | **p-Values below 0.05** |
| --- | --- | --- | --- | --- | --- |
| Caregiver Age | 0.17 | 0 | 0.12 | 0.11 | 1 |
| Caregiver Education | 0.01 | 0.27 | 0.27 | 0.46 | 1 |
| Caregiver Gender | 0.35 | 0.44 | 0.94 | 0.83 | 0 |
| Marital Status | 0.32 | 0.31 | 0.39 | 0.81 | 0 |
| Water Source | 0.71 | 0.76 | 0.29 | 1 | 0 |
| Electricity | 0.13 | 0 | 0.25 | 0.05 | 2 |
| Stove Ownership | 0.12 | 0.07 | 0.46 | 0 | 1 |
| Cellphone Ownership | 0.34 | 0.05 | 0.69 | 0 | 2 |
| Bicycle Ownership | 0.76 | 0.42 | 0.65 | 0.01 | 1 |
| Radio Ownership | 0.42 | 0.04 | 0.74 | 0.05 | 2 |
| Television Ownership | 0.04 | 0 | 0.12 | 0 | 3 |
| Internet via Phone | 0.52 | 0.48 | 0.82 | 0.29 | 0 |
| Household Members (count) | 0.34 | 0.13 | 0.02 | 0 | 2 |
| Polygamy | 0.6 | 0.21 | 0.56 | 0.96 | 0 |
| Earned Income | 0.97 | 0.25 | 0.51 | 0.49 | 0 |
| Community Savings Fund | 0.33 | 0.27 | 0.02 | 0.14 | 1 |
| Other Income Source | 0.36 | 0.03 | 0.77 | 0.57 | 1 |
| Child Left Home Alone | 0.03 | 0.26 | 0.89 | 0.01 | 2 |
| Household Monthly Income | 0.27 | 0.22 | 0.62 | 0 | 1 |
| Caregiver HIV Status | 0.14 | 0 | 0.28 | 0.34 | 1 |
| Region | 0.1 | 0.21 | 0.6 | 0 | 1 |
| Child Age (Months) | 0.71 | 0.14 | 0.74 | 0.06 | 0 |
| Child Gender | 0.38 | 0.86 | 0.33 | 0.59 | 0 |
| Child Relationship to Caregiver | 0.69 | 0.58 | 0.44 | 0.88 | 0 |
| Number of Siblings | 0.52 | 0 | 0.01 | 0.56 | 2 |
| Child HIV Status | 0.93 | 0.35 | 0.47 | 0.94 | 0 |
